# Variation and Disparities in Awareness of Myocardial Infarction Symptoms Among Adults in the United States

**DOI:** 10.1001/jamanetworkopen.2019.17885

**Published:** 2019-12-18

**Authors:** Shiwani Mahajan, Javier Valero-Elizondo, Rohan Khera, Nihar R. Desai, Ron Blankstein, Michael J. Blaha, Salim S. Virani, Bita A. Kash, William A. Zoghbi, Harlan M. Krumholz, Khurram Nasir

**Affiliations:** 1 Center for Outcomes Research and Evaluation, Yale New Haven Health, New Haven, Connecticut; 2 Section of Cardiovascular Medicine, Department of Medicine, Yale School of Medicine, New Haven, Connecticut; 3 Division of Cardiovascular Prevention and Wellness, Houston Methodist DeBakey Heart and Vascular Center, Houston, Texas; 4 Center for Outcomes Research, Houston Methodist Research Institute, Houston, Texas; 5 Division of Cardiology, University of Texas Southwestern Medical Center, Dallas; 6 Cardiovascular Imaging Program, Cardiovascular Division and Department of Radiology, Brigham and Women’s Hospital, Boston, Massachusetts; 7 The Johns Hopkins Ciccarone Center for Prevention of Cardiovascular Disease, Baltimore, Maryland; 8 Michael E DeBakey Veterans Affairs Medical Center, Houston, Texas; 9 Section of Cardiology, Baylor College of Medicine, Houston, Texas; 10 Department of Health Policy and Management, Yale School of Public Health, New Haven, Connecticut

## Abstract

**Importance:**

Prompt recognition of myocardial infarction symptoms is critical for timely access to lifesaving emergency cardiac care. However, patients with myocardial infarction continue to have a delayed presentation to the hospital.

**Objective:**

To understand the variation and disparities in awareness of myocardial infarction symptoms among adults in the United States.

**Design, Setting, and Participants:**

This cross-sectional study used data from the 2017 National Health Interview Survey among adult residents of the United States, assessing awareness of the 5 following common myocardial infarction symptoms among different sociodemographic subgroups: (1) chest pain or discomfort, (2) shortness of breath, (3) pain or discomfort in arms or shoulders, (4) feeling weak, lightheaded, or faint, and (5) jaw, neck, or back pain. The response to a perceived myocardial infarction (ie, calling emergency medical services vs other) was also assessed.

**Main Outcomes and Measures:**

Prevalence and characteristics of individuals who were unaware of myocardial infarction symptoms and/or chose not to call emergency medical services in response to these symptoms.

**Results:**

Among 25 271 individuals (13 820 women [51.6%; 95% CI, 50.8%-52.4%]; 17 910 non-Hispanic white individuals [69.9%; 95% CI, 68.2%-71.6%]; and 21 826 individuals [82.7%; 95% CI, 81.5%-83.8%] born in the United States), 23 383 (91.8%; 95% CI, 91.0%-92.6%) considered chest pain or discomfort a symptom of myocardial infarction; 22 158 (87.0%; 95% CI, 86.1%-87.8%) considered shortness of breath a symptom; 22 064 (85.7%; 95% CI, 84.8%-86.5%) considered pain or discomfort in arm a symptom; 19 760 (77.0%; 95% CI, 76.1%-77.9%) considered feeling weak, lightheaded, or faint a symptom; and 16 567 (62.6%; 95% CI, 61.6%-63.7%) considered jaw, neck, or back pain a symptom. Overall, 14 075 adults (53.0%; 95% CI, 51.9%-54.1%) were aware of all 5 symptoms, whereas 4698 (20.3%; 95% CI, 19.4%-21.3%) were not aware of the 3 most common symptoms and 1295 (5.8%; 95% CI, 5.2%-6.4%) were not aware of any symptoms. Not being aware of any symptoms was associated with male sex (odds ratio [OR], 1.23; 95% CI, 1.05-1.44; *P* = .01), Hispanic ethnicity (OR, 1.89; 95% CI, 1.47-2.43; *P* < .001), not having been born in the United States (OR, 1.85; 95% CI, 1.47-2.33; *P* < .001), and having a lower education level (OR, 1.31; 95% CI, 1.09-1.58; *P* = .004). Among 294 non-Hispanic black or Hispanic individuals who were not born in the United States, belonged to the low-income or lowest-income subgroup, were uninsured, and had a lower education level, 61 (17.9%; 95% CI, 13.3%-23.6%) were not aware of any symptoms. This group had 6-fold higher odds of not being aware of any symptoms (OR, 6.34; 95% CI, 3.92-10.26; *P* < .001) compared with individuals without these characteristics. Overall, 1130 individuals (4.5%; 95% CI, 4.0%-5.0%) chose a different response than calling emergency medical services in response to a myocardial infarction.

**Conclusions and Relevance:**

Many adults in the United States remain unaware of the symptoms of and appropriate response to a myocardial infarction. In this study, several sociodemographic subgroups were associated with a higher risk of not being aware. They may benefit the most from targeted public health initiatives.

## Key Points

**Question:**

What are the prevalence and characteristics of adults in the United States who remain unaware of the symptoms of and the appropriate response to a myocardial infarction?

**Findings:**

In this cross-sectional study of 25 271 US adults, 5.8% were not aware of any myocardial infarction symptoms, and 4.5% chose a different response than calling emergency medical services in response to these symptoms. These numbers were substantially higher in certain sociodemographic groups.

**Meaning:**

Many individuals in the United States remain unaware of the symptoms of and appropriate response to a myocardial infarction.

## Teaser

This cross-sectional study evaluates the prevalence and characteristics of US adults who are unaware of the symptoms of and appropriate response to myocardial infarction.

## Introduction

Although mortality rates among patients hospitalized for myocardial infarction (MI) have seen a decreasing trend, patients with MI continue to have a delayed presentation to the hospital, and a large number of them die before reaching the hospital.^1,2^ A critical aspect of lowering mortality associated with MI is ensuring timely access to lifesaving emergency cardiac care, for which prompt recognition of symptoms of a myocardial infarction (MI) and appropriate rapid emergency response are crucial.^3^

Previous studies from the United States have shown that, although awareness of MI symptoms has increased over the years, less than 50% of adults are aware of the 5 common symptoms (ie, chest pain or discomfort; shortness of breath; pain or discomfort in arms or shoulders; feeling weak, lightheaded, or faint; and jaw, neck, or back pain).^4,5,6,7,8^ Although Healthy People 2020 set targets to improve awareness of these common symptoms,^9^ there is little information on the prevalence and characteristics of individuals who are not aware of any symptoms. Additionally, previous studies on MI symptom awareness have focused on disparities across limited demographic subgroups (eg, age, sex, and race/ethnicity); however, the association of sociocultural factors (eg, education level, socioeconomic status [SES], insurance status, and immigration status) and the cumulative association of these potential risk factors with awareness remains largely unknown.^4,10^

Given that previous community interventions to improve awareness of symptoms and emergency medical service (EMS) use in MI have largely been unsuccessful,^11,12,13,14^ this information can help identify subgroups that are most in need of and may benefit from targeted public health awareness initiatives, which can subsequently reduce mortality and morbidity attributable to MI. Accordingly, we used nationally representative data to estimate awareness of MI symptoms among adults in the United States, characterizing sociodemographic groups, both individually and in combination, that were particularly at risk of not being aware of any symptoms.

## Methods

### Study Design and Population

We included 26 742 individuals aged 18 years and older, using data from the 2017 National Health Interview Survey (NHIS), which is an annual, cross-sectional, national, weighted survey that provides estimates on the noninstitutionalized US population using multistage sampling.^15^ Additional details of the NHIS survey are provided in the eMethods in the Supplement. We excluded 1471 participants because of missing information on awareness of MI symptoms (eFigure 1 in the Supplement). This study was exempt from review by the Yale University institutional review board committee because NHIS data are publicly available and deidentified. The study was reported in accordance with the Strengthening the Reporting of Observational Studies in Epidemiology (STROBE) reporting guideline.

### Awareness of MI Symptoms

Awareness was assessed by an individual’s responses to the question, “Which of the following would you say are the symptoms that someone may be having a heart attack?”: (1) chest pain or discomfort; (2) shortness of breath; (3) pain or discomfort in arms or shoulders; (4) feeling weak, lightheaded, or faint; and (5) jaw, neck, or back pain. We studied responses individually, then divided them into 4 mutually exclusive subgroups based on the number of symptoms an individual was aware of, as follows: (1) none of the symptoms, (2) 1 to 2 symptoms, (3) 3 to 4 symptoms, and (4) all 5 symptoms. We also assessed the awareness of the 3 most common symptoms (ie, chest pain or discomfort; shortness of breath; and pain or discomfort in arms or shoulders) separately.

### Response to a Perceived MI

We assessed the prevalence of adults who were aware of the need to access immediate emergency care by calling EMS in response to a perceived MI by their response to the question, “What is best thing to do when someone is having a heart attack?” Responses included call 9-1-1 or another emergency number, advise them to drive to the hospital, advise them to call their physician, call spouse or family member, and other. We studied all responses individually, then dichotomized the responses to calling 9-1-1 or another emergency number vs all other options.

### Independent Variables

Other variables included in this study were age (ie, 18-39 years, 40-64 years, or ≥65 years), sex (ie, male or female), race/ethnicity (ie, non-Hispanic white, non-Hispanic black, or Hispanic), SES (based on family income as a percentage of the federal poverty limit from the US Census Bureau and classified as high income [≥400%], middle income [200% to <400%], low income [125% to <200%], and lowest income [<125%]), education level (ie, ≥some college or ≤high school), insurance status (ie, public, private, or uninsured), geographic region (ie, Northeast, Midwest, South, or West), and immigration status (based on geographic place of birth and classified as US-born or non-US-born). For non-US-born individuals, we also collected information on their time in the United States (ie, <10 years vs ≥10 years) and English proficiency (ie, speaks English well or very well vs does not speak English well or at all). English proficiency was measured directly, and in cases where the interviewee did not speak English well (<1.5%), a proxy was used to answer the survey questions.

### Statistical Analysis

The NHIS uses complex sampling techniques to select the sample. After adjusting for nonresponse, age, sex, and race/ethnicity (based on the population estimates produced by the US Census Bureau), final person-level weights are created, which can then be used to provide national estimates. We described the survey-weighted proportions (with Rao-Scott χ^2^) of awareness for each of the 5 symptoms individually, the distribution of overall awareness (from 0 to 5), and the awareness of the 3 most common symptoms across different sociodemographic characteristics. Next, we assessed the association of these characteristics with not being aware of any MI symptoms using unadjusted and adjusted survey-specific logistic regression and multinomial regression models. Logistic regression was used to evaluate dichotomous outcome variables (eg, being aware of none vs any MI symptoms), while multinomial regression was used to study categorical outcome variables (eg, being aware of 0 vs 1 vs 2 vs 3 vs 4 vs all 5 MI symptoms). Explanatory variables included age, sex, race/ethnicity, immigration status, education level, SES, insurance status, and region. We also created a composite score using race/ethnicity (non-Hispanic white vs non-Hispanic black and Hispanic), immigration status (US-born vs non-US-born), education level (≥some college vs ≤high school), SES (high or middle income vs low or lowest income), and insurance status (insured vs uninsured) to study the cumulative association of these factors with awareness of MI symptoms. We also assessed the proportion of individuals who chose a different response than calling EMS as a reaction to a perceived MI, both overall and by awareness of MI symptoms. We identified individual characteristics associated with not calling EMS in response to a MI, using unadjusted and adjusted survey-specific logistic regression models.

We considered *P* < .05 statistically significant a priori for all analyses in our study, and all tests were 2-tailed. All analyses were performed using Stata version 13.0 (StataCorp) and accounted for the survey design of the NHIS, including sampling weights, to ensure that our results were nationally representative.

## Results

### Population Characteristics

Our study population included 25 271 individuals corresponding to more than 233.4 million adults in 2017; 13 820 (51.6%; 95% CI, 50.8%-52.4%) were women; 17 910 (69.9%; 95% CI, 68.2%-71.6%) were non-Hispanic white individuals; and 21 826 (82.7%; 95% CI, 81.5%-83.8%) were born in the United States (Table 1). A total of 7446 participants (28.3%; 95% CI, 27.0%-29.8%), representing an estimated 62.1 million individuals, were part of the low- or lowest-income subgroup, and 8683 participants (35.2%; 95% CI, 34.1%-36.3%), representing an estimated 81.8 million individuals, had an education level of high school or less. Most individuals had private insurance (12 745 [55.9%; 95% CI, 54.8%-57.0%]), followed by public insurance (10 274 [34.4%; 95% CI, 33.4%-35.4%]) and no insurance (2173 [9.7%; 95% CI, 9.1%-10.4%]).

**Table 1.** Characteristics of Study Participants

Characteristic	No. (N = 25 271)	Weighted % (95% CI)	Estimated US Population, No. (N = 233 427 109)
Age, y			
18-39	8198	38.46 (37.51-39.41)	89 771 938
40-64	10 304	41.90 (41.03-42.78)	97 811 097
≥65	6769	19.64 (19.01-20.29)	45 844 074
Sex			
Men	11 451	48.43 (47.65-49.22)	113 053 335
Women	13 820	51.57 (50.78-52.35)	120 373 774
Race/ethnicity			
Non-Hispanic white	17 910	69.93 (68.18-71.62)	151 775 038
Non-Hispanic black	2782	13.11 (12.04-14.25)	28 445 310
Hispanic	3010	16.97 (15.52-18.51)	36 822 570
Immigration status			
US-born	21 826	82.70 (81.51-83.83)	192 932 915
Non-US-born	3428	17.30 (16.17-18.49)	40 361 715
Education			
≥Some college	16 517	64.84 (63.71-65.95)	150 806 910
≤High school	8683	35.16 (34.05-36.29)	81 780 160
Family income subgroup			
High	9604	43.14 (41.85-44.44)	94 485 977
Middle	6737	28.48 (27.64-29.34)	62 370 753
Low	4218	16.72 (16.02-17.45)	36 621 219
Lowest	3228	11.65 (10.95-12.39)	25 522 093
Insurance			
Private	12 745	55.89 (54.83-56.95)	129 835 329
Public	10 274	34.38 (33.39-35.38)	79 855 830
Uninsured	2173	9.73 (9.09-10.40)	22 593 891
Region			
Northeast	4103	18.36 (16.80-20.03)	42 860 199
Midwest	6036	21.92 (20.71-23.17)	51 159 938
South	9366	36.42 (34.40-38.49)	85 010 955
West	5766	23.30 (21.59-25.10)	54 396 017

### Awareness of MI Symptoms

In this nationally representative adult population, most individuals (23 383 [91.8%; 95% CI, 91.0%-92.6%]) considered chest pain or discomfort a MI symptom, followed by shortness of breath (22 158 [87.0%; 95% CI, 86.1%-87.8%]), pain or discomfort in arms or shoulders (22 064 [85.7%; 95% CI, 84.8%-86.5%]), feeling weak, lightheaded, or faint (19 760 [77.0%; 95% CI, 76.1%-77.9%]), and jaw, neck, or back pain (16 567 [62.6%; 95% CI, 61.6%-63.7%]) (eTable 1 in the Supplement). Awareness of symptoms was significantly higher among individuals who were non-Hispanic white, born in the United States, had higher education levels, belonged to the high-income or middle-income subgroup, and had private insurance compared with individuals who were non-Hispanic black or Hispanic, were not born in the United States, had lower education levels, belonged to the low-income or lowest-income subgroup, and were uninsured (eTable 1 in the Supplement). For example, 16 959 of all non-Hispanic white participants (94.4%; 95% CI, 93.5%-95.1%) were aware that chest pain or discomfort is a symptom of MI, while 2529 of all Hispanic participants (84.8%; 95% CI, 83.0%-86.4%) were aware of this symptom; more individuals in the high-income subgroup than those in the lowest-income subgroup were aware that jaw, neck, or back pain is a symptom of MI (6694 [67.1%; 95% CI, 65.6%-68.5%] vs 1853 [55.3%; 95% CI, 52.7%-57.8%]) (eTable 1 in the Supplement).

Overall, 14 075 individuals (53.0%; 95% CI, 51.9%-54.1%), representing 123.7 million adults, were aware of all 5 MI symptoms, whereas 4698 (20.3%; 95% CI, 19.4%-21.3%), representing 47.5 million adults, were not aware of the 3 most common symptoms and 1295 (5.8%; 95% CI, 5.2%-6.4%), representing 13.5 million adults, were not aware of any symptoms (eTable 2 and eTable 3 in the Supplement). Awareness of different numbers of MI symptoms (ranging from 0-5) varied substantially across sociodemographic subgroups (eFigure 2 in the Supplement). The proportion of individuals not aware of any of the symptoms was higher among non-Hispanic black and Hispanic individuals than non-Hispanic white individuals (164 [6.6%; 95% CI, 5.3%-8.2%] and 331 [10.5%; 95% CI, 9.1%-12.0%] vs 653 [4.0%; 95% CI, 3.4%-4.7%]; *P* < .001), among individuals not born in the United States than those born in the United States (418 [11.9%; 95% CI, 10.5%-13.4%] vs 877 [4.5%; 95% CI, 3.9%-5.2%]; *P* < .001), among individuals with lower education levels than those with higher education levels (623 [8.1%; 95% CI, 7.2%-9.0%] vs 667 [4.5%; 95% CI, 3.9%-5.3%]; *P* < .001), among individuals belonging to the low-income and lowest-income subgroups than those belonging to the high-income and middle-income subgroups (222 [8.1%; 95% CI, 6.8%-9.5%] and 285 [7.8%; 95% CI, 6.7%-9.0%] vs 339 [4.0%; 95% CI, 3.3%-4.8%] and 334 [5.8%; 95% CI, 4.9%-6.8%]; *P* < .001), among individuals with no insurance than those with public and private insurance (211 [9.9%; 95% CI, 8.4%-11.8%] vs 534 [6.0%; 95% CI, 5.3%-6.8%] and 547 [4.9%; 95% CI, 4.2%-5.8%]; *P* < .001), and among individuals living in the South than those living in the Midwest (588 [7.0%; 95% CI, 5.7%-8.5%] vs 245 [4.4%; 95% CI, 3.6%-5.5%]; *P* < .001) (eFigure 3 in the Supplement). The proportion of individuals who were not aware of the 3 most common symptoms was higher across similar subgroups (eg, Hispanic vs non-Hispanic white individuals, 936 [31.7%; 95% CI, 29.4%-34.0%] vs 2541 [14.8%; 95% CI, 13.9%-15.7%]; *P* < .001; non-US-born vs US-born individuals, 1227 [36.4%; 95% CI, 34.2%-38.7%] vs 3469 [17.0%; 95% CI, 16.1%-17.9%]; *P* < .001; lowest-income vs high-income subgroup, 879 [29.4%; 95% CI, 26.9%-32.1%] vs 1325 [15.3%; 95% CI, 14.2%-16.5%]; *P* < .001) (eTable 3 in the Supplement).

In a subanalysis among individuals not born in the United States, we found that those who did not have good English proficiency were less likely to be aware of all 5 symptoms than those who had good English proficiency (298 [95% CI, 34.6%; 30.4%-39.1%] vs 1072 [40.2%; 95% CI, 37.6%-42.9%]; *P* < .001), and those who had been in the United States for less than 10 years were less likely to be aware of all 5 symptoms than those had been in the United States for 10 or more years (199 [29.4%; 95% CI, 25.1%-34.2%] vs 1137 [40.5%; 95% CI, 37.8%-43.2%]; *P* < .001). Similarly, those who did not have good English proficiency were more likely than those who had good English proficiency to be aware of none of the symptoms (185 [19.6%; 95% CI, 16.4%-23.2%] vs 233 [9.1%; 95% CI, 7.7%-10.7%]; *P* < .001), and those who had been in the United States for less than 10 years were more likely than those who had been in the United States for 10 or more years to be aware of none of the symptoms (98 [14.8%; 95% CI, 11.5%-18.7%] vs 315 [11.2%; 95% CI, 9.8%-12.8%]; *P* < .001) (eFigure 4 in the Supplement).

### Sociodemographic Characteristics Associated With Lack of Awareness

**Figure 1.**
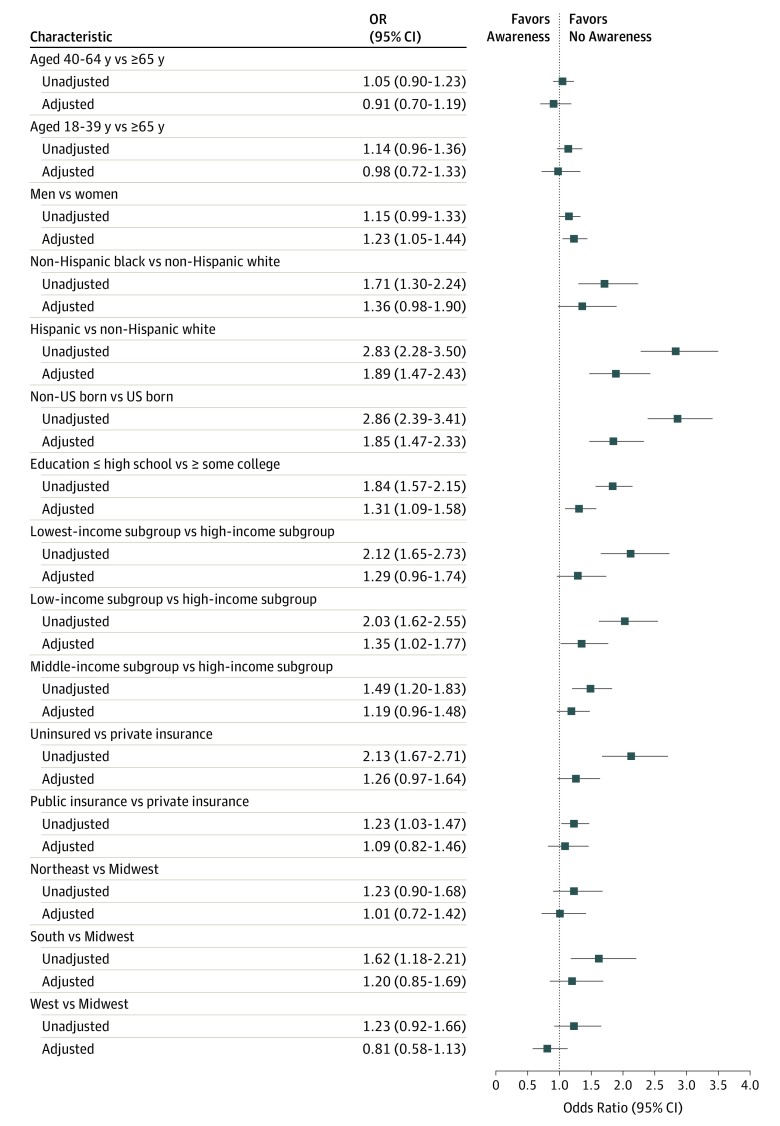
Unadjusted and Risk-Adjusted Associations of Sociodemographic Characteristics With Not Being Aware of Any Symptoms of a Myocardial Infarction Results show odds ratios (ORs) with 95% CI calculated using logistic regression. Adjusted model includes all variables presented in the figure.

Overall, several individual characteristics were associated with not being aware of any MI symptoms (Figure 1). In an unadjusted model, we found that higher odds of not being aware of any symptoms were associated with black race (odds ratio [OR], 1.71; 95% CI, 1.30-2.24; *P* < .001) and Hispanic ethnicity (OR, 2.83; 95% CI, 2.28-3.50; *P* < .001) compared with non-Hispanic white race/ethnicity, not having been born in the United States (OR, 2.86; 95% CI, 2.39-3.41; *P* < .001) compared with being born in the United States, lower education levels (OR, 1.84; 95% CI, 1.57-2.15; *P* < .001) compared with higher education levels, the low-income (OR, 2.03; 95% CI, 1.62-2.55; *P* < .001) or lowest-income (OR, 2.12; 95% CI, 1.65-2.73; *P* < .001) subgroup compared with the highest-income subgroup, public (OR, 1.23; 95% CI, 1.03-1.47; *P* = .02) or no (OR, 2.13; 95% CI, 1.67-2.71; *P* < .001) insurance compared with private insurance, and living in the South (OR, 1.62; 95% CI, 1.18-2.21; *P* = .003) compared with living in the Midwest. When we adjusted for known confounders, we found that higher odds of not being aware of any symptoms were associated with male sex (OR, 1.23; 95% CI, 1.05-1.44; *P* = .01) compared with female sex, Hispanic ethnicity (OR, 1.89; 95% CI, 1.47-2.43; *P* < .001) compared with non-Hispanic white race/ethnicity, not being born in the United States (OR, 1.85; 95% CI, 1.47-2.33; *P* < .001) compared with being born in the United States, and lower education levels (OR, 1.31; 95% CI, 1.09-1.58; *P* = .004) compared with higher education levels (Figure 1).

Using multinomial regression (adjusting for the statistically significant factors from the previous logistic regression), we found that there was a stepwise higher likelihood of not being aware as the aggregate number of symptoms grew. For example, compared with individuals born in the United States, those not born in the United States were 30% (relative risk ratio [RRR], 0.70; 95% CI, 0.53-0.93; *P* = .01) less likely to be aware of 3 symptoms, 52% (RRR, 0.48; 95% CI, 0.37-0.62; *P* < .001) less likely to be aware of 4 symptoms, and 54% (RRR, 0.46; 95% CI, 0.36-0.58; *P* < .001) less likely to be aware of 5 symptoms of a MI compared with being aware of no symptoms. Similar trends in association were seen across other subgroups (eg, individuals with lower education levels had RRRs of 0.73 [95% CI, 0.61-0.89; *P* = .001] for being aware of 4 symptoms and 0.68 [95% CI, 0.56-0.82; *P* < .001] for being aware of 5 symptoms) (eTable 4 in the Supplement).

### Cumulative Association of Sociodemographic Factors With Awareness

**Figure 2.**
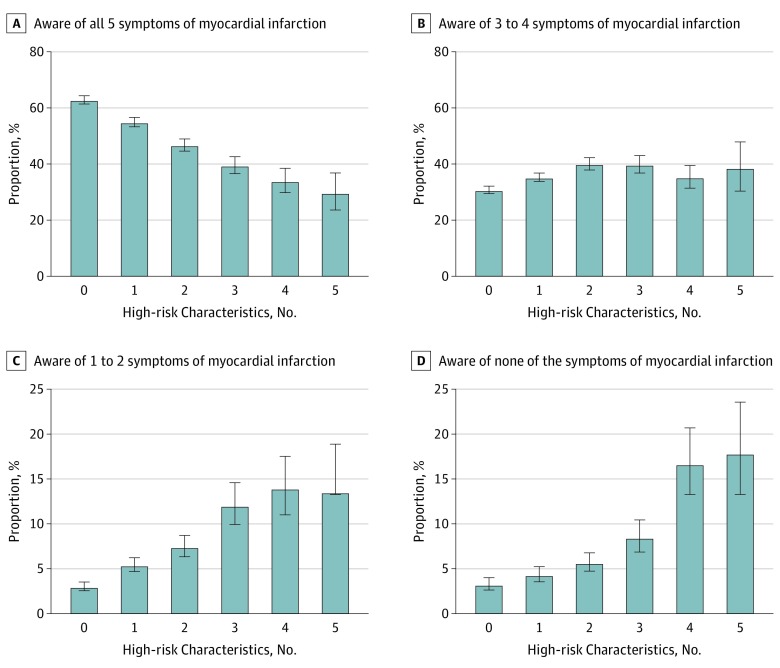
Proportion of Individuals Aware of Different Number of Myocardial Infarction Symptoms by Number of High-Risk Characteristics High-risk characteristics include non-Hispanic black or Hispanic race/ethnicity, non-US-born immigrant status, low-income or lowest-income subgroup, uninsured, and high school or lower education level. Error bars indicate 95% CIs.

We evaluated 5 variables (ie, race/ethnicity, immigration status, education, income, and insurance status) associated with the greatest risk of not being aware of any MI symptoms and examined their combined association with awareness. Compared with the reference group with no high-risk characteristics (8793 white and US-born individuals who belonged to the middle-income or high-income subgroup, had insurance, and had a higher education level), those with 1, 2, 3, 4, and 5 high-risk characteristics had a stepwise decrease in awareness (Figure 2). Among 294 individuals with all 5 high-risk characteristics (representing 3.7 million adults in the United States), 88 (29.8%; 95% CI, 23.6%-36.8%) were aware of all 5 symptoms, compared with 5688 (62.9%; 95% CI, 61.5%-64.4%) in the reference group. Moreover, 61 individuals (17.9%; 95% CI, 13.3%-23.6%) with all 5 high-risk characteristics (representing approximately 664 143 adults) were not aware of a single MI symptom compared with 253 individuals (3.3%; 95% CI, 2.6%-4.0%) in the reference group (Figure 2). Using logistic regression analysis, we found that, compared with the reference group, those with all 5 high-risk characteristics had more than 6-fold higher odds of not being aware of any symptoms (OR, 6.34; 95% CI, 3.92-10.26; *P* < .001) (Table 2).

**Table 2.** Odds of Not Being Aware of Any Myocardial Infarction Symptoms Based on the Number of High-Risk Characteristics

High-Risk Characteristic, No.^a^	Unadjusted Model		Adjusted Model^b^	
	OR (95% CI)	*P* Value	OR (95% CI)	*P* Value
0	1 [Reference]	NA	1 [Reference]	NA
1	1.34 (1.07-1.69)	.01	1.33 (1.06-1.68)	.01
2	1.79 (1.37-2.33)	<.001	1.75 (1.33-2.31)	<.001
3	2.76 (2.02-3.76)	<.001	2.69 (1.96-3.70)	<.001
4	5.94 (4.31-8.19)	<.001	5.89 (4.23-8.21)	<.001
5	6.46 (4.13-10.10)	<.001	6.34 (3.92-10.26)	<.001

Abbreviations: NA, not applicable; OR, odds ratio.

^a^ High-risk characteristics include non-Hispanic black or Hispanic race/ethnicity, non-US-born immigrant status, low-income or lowest-income subgroup, uninsured, and high school or lower education level.

^b^ Model adjusted for age, sex, and region.

### Response to a Perceived MI

Overall, 1130 individuals (4.5%; 95% CI, 4.0%-5.0%), representing 10.4 million adults, chose a different response than calling EMS in response to a perceived MI (eTable 5 in the Supplement). The proportion was significantly higher among individuals who were 65 years or older than among those who were aged 18 to 39 years (410 [5.8%] vs 285 [4.0%]; *P* = .001), men than women (540 [4.9%] vs 590 [4.1%]; *P* = .02), those who were not born in the United States than those who were born in the United States (197 [5.9%] vs 933 [4.2%]; *P* = .005), those who had a lower education level than those with a higher education level (455 [5.5%] vs 671 [3.9%]; *P* = .001), those belonging to the low-income and lowest-income subgroups than those belonging to the middle-income or highest-income subgroups (386 [11.3%] vs 653 [7.9%]; *P* < .001), and those with no insurance than those with private insurance (125 [6.8%] vs 436 [3.5%]; *P* < .001) (eTable 6 in the Supplement). In analysis using logistic regression, being 65 years or older (OR, 1.63; 95% CI, 1.22-2.19; *P* = .001) and uninsured (OR, 1.59; 95% CI, 1.19-2.12; *P* = .001) had the strongest associations with not calling EMS in response to a perceived MI compared with being younger than 65 years and having private insurance, respectively (eTable 7 in the Supplement).

**Figure 3.**
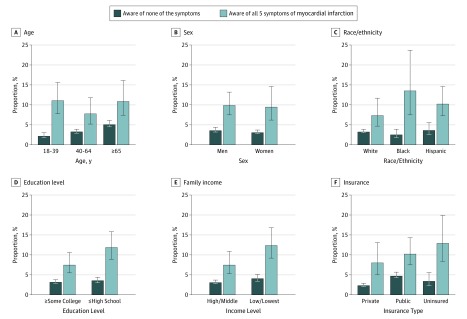
Proportion of Individuals Who Chose a Response Other Than Calling Emergency Medical Services in Response to a Perceived Myocardial Infarction, by Sociodemographic Characteristics and Awareness of Myocardial Infarction Symptoms Error bars indicate 95% CIs.

In assessing response to a MI by awareness of MI symptoms, we found that 115 adults (9.8%; 95% CI, 7.7%-12.4%) among those who were not aware of any symptoms (representing 1.3 million individuals) chose a different response than calling EMS, compared with 538 adults (3.4%; 95% CI, 3.1%-3.9%) among those who were aware of all 5 symptoms of a MI (representing approximately 4.3 million adults) (eTable 8 in the Supplement). These differences were consistently seen across all sociodemographic subgroups. For example, among individuals with an education level of high school or less who were aware of none of the symptoms, 69 (12.0%; 95% CI, 8.9%-15.8%) chose a different response than calling EMS compared with 176 (3.6%; 95% CI, 3.0%-4.4%) individuals with an education level of high school or less who were aware of all 5 symptoms (*P* < .001). Among individuals who belonged to the low-income or lowest-income subgroup and were aware of none of the symptoms, 55 (12.5%; 95% CI, 9.2%-16.8%) chose a different response than calling EMS compared with 155 (4.2%; 95% CI, 3.4%-5.1%) who belonged to the low-income or lowest-income subgroups and were aware of all 5 symptoms (*P* < .001) (Figure 3).

## Discussion

In this nationally representative cross-sectional study, we found that nearly 6% of individuals, or an estimated 13.5 million adults nationally, were not aware of a single symptom of a MI and nearly 1 in 12 individuals, or an estimated 19.1 million adults nationally, did not consider chest pain or discomfort a MI symptom. These numbers were substantially higher for individuals who were non-Hispanic black or Hispanic, were not born in the United States, had lower education levels, were uninsured, and belonged to the low-income and lowest-income subgroups. Among individuals having all these characteristics, 1 in 5 were not aware of any symptom of a MI. Moreover, nearly 4.5% of individuals, or an estimated 10.4 million adults nationally, chose a different response than immediately calling EMS on suspicion of a MI, and this proportion was more than double (9.8%) among adults who were not aware of any MI symptoms.

Our study extends the previous literature on awareness of MI symptoms in several ways. First, most previous studies describing the awareness of MI symptoms have focused on individuals who were aware of all 5 symptoms.^4,6,8,16,17^ However, we focused on individuals who were not aware of any or the most common symptoms and identified subgroups that were most in need of and may benefit the most from targeted public health awareness initiatives. Studies have reported a 10.1% increase in awareness of all 5 symptoms between 2008 and 2014, with 47.2% adults in the United States being aware of all 5 symptoms in 2014.^4^ Our results not only showed a small increase in awareness of all 5 symptoms since 2014 but also suggest that, even today, millions of individuals in the United States remain unaware of the most critical symptoms of a MI (eg, chest pain) and, therefore, are at a high risk of adverse outcomes after an MI.

Second, to our knowledge, this study is the first to describe awareness rates across such diverse sociodemographic subgroups based on SES, insurance status, and immigration status. We found significant disparities across subgroups based on age, race/ethnicity, and education level, which were consistent with previous reports on awareness^4,10,16,18,19^ and, additionally, identified non-US-born individuals, uninsured individuals, and individuals from the low-income and lowest-income subgroups as high-risk subgroups for not being aware of any symptoms.

Third, to our knowledge, this is the first report studying the awareness of MI symptoms among immigrants and describing the association of acculturation factors (eg, English proficiency and duration of US residence) with awareness. We found that nearly 1 in 8 (12%) of the estimated 5 million non-US-born individuals were not aware of any symptoms and that acculturation factors had a significant association with awareness among immigrants. Given the increasing number of individuals in the United States who were born in other countries and the low symptom awareness rates among these individuals,^20^ public health professionals may need to tailor awareness campaigns according to these individuals’ linguistic and cultural needs.

Fourth, to our knowledge, our study is the first to describe the cumulative association of the potential high-risk characteristics (ie, non-Hispanic black or Hispanic race/ethnicity, non-US-born, low income, uninsured, lower education level) with awareness. We reported a stepwise increase in the proportion of individuals who were not aware of any MI symptoms as the number of these high-risk characteristics increased. Among individuals with all 5 high-risk characteristics, nearly 1 in 5 individuals were not aware of any of the symptoms. As such, our findings underscore the importance of targeting public health initiatives toward these socioeconomically disadvantaged groups to improve awareness and subsequently reduce the mortality associated with MI.

Finally, our assessment of the use of EMS in response to a perceived MI suggests that, although the use of EMS has increased from that previously reported in the literature (91.8% in 2008 and 93.4% in 2014),^8^ millions of individuals continued to choose a different response than immediately calling EMS. As expected, individuals who were unaware of the symptoms were also more likely to not call EMS; however, a significant number of adults with optimal symptom awareness also chose to not call EMS. Some possible explanations for this could be denial of symptoms, misattribution to symptoms to a noncardiac cause, perceived loss of control and ability to act, self-treatment strategies, fear or embarrassment of being wrong, and concerns about cost.^4,10,21,22,23^ Given that early intervention in patients with MI is crucial to limit ischemic damage, prompt recognition of MI symptoms and rapid decision to seek care can reduce delays from symptom-onset to hospital presentation and improve survival. As such, it is critical to not only improve awareness of warning signs of a MI and the importance of early access to medical care but also to better understand and address the barriers that prevent individuals from accessing emergency medical care.

The American Heart Association, the US Department of Health and Human Services, the National Heart, Lung, and Blood Institute, and the US Centers for Disease Control and Prevention have made substantial efforts to improve awareness of MI symptoms, such as the Go Red for Women and Go Red Por Tu Corazon (ie, Go Red for your Heart, which targets Spanish-speaking women), Make the Call, Don’t Miss a Beat, The Heart Truth, and WISEWOMAN campaigns, respectively.^24,25,26,27,28^ While most of these initiatives are directed to women, our study found a nearly 10% higher awareness of all 5 MI symptoms and better use of EMS among women than men, which could be a reflection of the successful reach of these campaigns, although efforts to increase awareness of cardiovascular disease among women are still warranted.^29^

Disparities in awareness and response to MI symptoms found in our study corresponded closely with the disparities seen in delays in hospital presentation and outcomes after MI.^30,31,32,33,34,35^ Racial and ethnic minorities have been shown to have longer delay times than non-Hispanic white individuals.^31^ Similarly, individuals with lower SES and greater financial concerns have been shown to have a delayed presentation to the hospital, although this could be related to issues with access to care.^32^ It has been shown that focusing on the seriousness of the situation and increasing awareness among specific population subgroups could be useful in reducing prehospital delays.^4,36,37^ As such, recognizing the subgroups that are at the highest risk of being unaware of MI symptoms is germane to the current debate regarding diminishing treatment delays for individuals experiencing a MI and can help better design health care policies and/or campaigns specifically tailored for them.^4^

### Limitations

This study has limitations. First, our assessment of awareness of MI symptoms was based on an arbitrary list, and while the most prevalent symptoms were listed, presentation of a MI may not be limited to these symptoms. Nevertheless, we showed that millions of individuals were unaware of even these most common symptoms of a MI. Second, not all MI symptoms included in this study should be weighted equally because some symptoms (eg, chest pain or discomfort) may be more easily identifiable than others. Therefore, although we provided the distribution of awareness of all MI symptoms and a composite score, we chose to focus our analyses on those who were not aware of any symptoms. Third, our assessment of MI symptom awareness was based on a set of closed-ended questions (ie, yes or no) that may bias responses, and offering of a set of symptoms could have led to an overestimation of the awareness rates. As such, the actual awareness rates may be even lower than those reported in our study. Fourth, although we studied and adjusted for the most important sociodemographic variables, MI awareness can inherently be driven by personal or familial exposure, which we were not able to assess because NHIS does not include this information. Fifth, because of the low sample size of Asian and other racial/ethnic groups, we could evaluate disparities only among the non-Hispanic white, non-Hispanic black, and Hispanic subgroups. Sixth, we could have overestimated the proportion of individuals choosing to call the EMS in response to a perceived MI because of a social desirability bias in responding; survey respondents may tend to answer questions in a manner that will be viewed favorably by the interviewer. Despite that, millions of individuals chose a different response than immediately calling EMS and could benefit from increasing awareness regarding the importance of early access to medical care.

## Conclusions

Our study found that 53% of US adults in this study, representing 123.7 million adults in the United States, were aware of all 5 MI symptoms, and nearly 6% of individuals in our study, or an estimated 13.5 million adults nationally, were not aware of a single symptom of a MI. Additionally, significant sociodemographic disparities were seen in both the awareness of and appropriate response to MI symptoms. These findings highlight the need for targeted educational campaigns to not only improve awareness of MI symptoms but also emphasize the importance of early access to emergency medical care across all sociodemographic subgroups.

## Notes

Supplement. **eMethods. **Brief Description of the Survey Design for the National Health Interview Survey **eFigure 1. **Selection of Study Participants **eFigure 2. **Distribution of Awareness of Myocardial Infarction Symptoms by Sociodemographic Characteristics **eFigure 3.** Weighted Proportion of Individuals Who Were Not Aware of Any Myocardial Infarction Symptoms by Sociodemographic Characteristics **eFigure 4. **Distribution of Awareness of Myocardial Infarction Symptoms and the Weighted Proportion of Non-US-Born Individuals Who Were Not Aware of Any Myocardial Infarction Symptoms, by English Proficiency and Years in the United States **eTable 1. **Awareness of Individual Symptoms of a Myocardial Infarction, by Sociodemographic Characteristics **eTable 2. **Awareness of Symptoms of a Myocardial Infarction (0 to 5), by Sociodemographic Characteristics **eTable 3. **Proportion of Individuals Who Were Not Aware of the 3 Most Common Symptoms of a Myocardial Infarction, by Sociodemographic Characteristics **eTable 4. **Association Between Population Characteristics and Awareness of Myocardial Infarction Symptoms Using Multinomial Regression Analysis **eTable 5. **Distribution of Different Responses to Assessment of Emergency Response to Suspicion of Myocardial Infarction **eTable 6. **Proportion of Individuals Who Chose a Response Other Than Calling Emergency Medical Services in Response to Suspicion of a Myocardial Infarction, by Sociodemographic Characteristics **eTable 7. **Association of Sociodemographic Characteristics With Choosing a Response Other Than Calling Emergency Medical Services on Suspicion of a Myocardial Infarction Using Logistic Regression **eTable 8. **Proportion of Individuals Who Chose a Response Other Than Calling Emergency Medical Services in Response to Suspicion of a Myocardial Infarction, by Sociodemographic Characteristics and Awareness of Myocardial Infarction Symptoms
