# Supplementary material for: Variation and Disparities in Awareness of Myocardial Infarction Symptoms Among Adults in the United States
Source: JAMA Netw Open. 2019 Dec 18;2(12):e1917885. doi: 10.1001/jamanetworkopen.2019.17885 (PMC6991230; doi:10.1001/jamanetworkopen.2019.17885)
Supplement: Supplement. — eMethods. Brief Description of the Survey Design for the National Health Interview Survey eFigure 1. Selection of Study Participants eFigure 2. Distribution of Awareness of Myocardial Infarction Symptoms by Sociodemographic Characteristics eFigure 3. Weighted Proportion of Individuals Who Were Not Aware of Any Myocardial Infarction Symptoms by Sociodemographic Characteristics eFigure 4. Distribution of Awareness of Myocardial Infarction Symptoms and the Weighted Proportion of Non-US-Born Individuals Who Were Not Aware of Any Myocardial Infarction Symptoms, by English Proficiency and Years in the United States eTable 1. Awareness of Individual Symptoms of a Myocardial Infarction, by Sociodemographic Characteristics eTable 2. Awareness of Symptoms of a Myocardial Infarction (0 to 5), by Sociodemographic Characteristics eTable 3. Proportion of Individuals Who Were Not Aware of the 3 Most Common Symptoms of a Myocardial Infarction, by Sociodemographic Characteristics eTable 4. Association Between Population Characteristics and Awareness of Myocardial Infarction Symptoms Using Multinomial Regression Analysis eTable 5. Distribution of Different Responses to Assessment of Emergency Response to Suspicion of Myocardial Infarction eTable 6. Proportion of Individuals Who Chose a Response Other Than Calling Emergency Medical Services in Response to Suspicion of a Myocardial Infarction, by Sociodemographic Characteristics eTable 7. Association of Sociodemographic Characteristics With Choosing a Response Other Than Calling Emergency Medical Services on Suspicion of a Myocardial Infarction Using Logistic Regression eTable 8. Proportion of Individuals Who Chose a Response Other Than Calling Emergency Medical Services in Response to Suspicion of a Myocardial Infarction, by Sociodemographic Characteristics and Awareness of Myocardial Infarction Symptoms [file jamanetwopen-2-e1917885-s001.pdf]

Supplementary Online Content

Mahajan S, Valero-Elizondo J, Khera R, et al. Variation and disparities in awareness of myocardial infarction symptoms among adults in the United States. *JAMA Netw Open*. 2019;2(12):e1917885. doi:10.1001/jamanetworkopen.2019.17885

eMethods. Brief Description of the Survey Design for the National Health Interview Survey

eFigure 1. Selection of Study Participants

eFigure 2. Distribution of Awareness of Myocardial Infarction Symptoms by Sociodemographic Characteristics

eFigure 3. Weighted Proportion of Individuals Who Were Not Aware of Any Myocardial Infarction Symptoms by Sociodemographic Characteristics

eFigure 4. Distribution of Awareness of Myocardial Infarction Symptoms and the Weighted Proportion of Non-US-Born Individuals Who Were Not Aware of Any Myocardial Infarction Symptoms, by English Proficiency and Years in the United States

eTable 1. Awareness of Individual Symptoms of a Myocardial Infarction, by Sociodemographic Characteristics

eTable 2. Awareness of Symptoms of a Myocardial Infarction (0 to 5), by Sociodemographic Characteristics

eTable 3. Proportion of Individuals Who Were Not Aware of the 3 Most Common Symptoms of a Myocardial Infarction, by Sociodemographic Characteristics

eTable 4. Association Between Population Characteristics and Awareness of Myocardial Infarction Symptoms Using Multinomial Regression Analysis

eTable 5. Distribution of Different Responses to Assessment of Emergency Response to Suspicion of Myocardial Infarction

eTable 6. Proportion of Individuals Who Chose a Response Other Than Calling Emergency Medical Services in Response to Suspicion of a Myocardial Infarction, by Sociodemographic Characteristics

eTable 7. Association of Sociodemographic Characteristics With Choosing a Response Other Than Calling Emergency Medical Services on Suspicion of a Myocardial Infarction Using Logistic Regression

© 2019 Mahajan S et al. *JAMA Network Open.*

eTable 8. Proportion of Individuals Who Chose a Response Other Than Calling Emergency Medical Services in Response to Suspicion of a Myocardial Infarction, by Sociodemographic Characteristics and Awareness of Myocardial Infarction Symptoms

This supplementary material has been provided by the authors to give readers additional information about their work.

© 2019 Mahajan S et al. *JAMA Network Open.*

eMethods. Brief Description of the Survey Design for the National Health Interview Survey

National Health Interview Survey (NHIS) is an annual, cross-sectional national weighted survey that provides estimates on the noninstitutionalized US population using multistage sampling.

Response rates
The participation rates for NHIS are actually very high. The conditional response rate for the Sample Adult component was 80.7%, which was calculated by dividing the number of completed Sample Adult interviews (n=26,742) by the total number of eligible sample adults (n=33,143).

Weighting
The final Sample Adult Weight includes design, ratio, nonresponse and post-stratification adjustments for sample adults. National estimates of all sample adult variables can be made using these weights.

Use of Proxy
In the NHIS, sample adults generally respond for themselves, although in a small number of cases, proxy responses are allowed if the selected adult had a physical or mental condition prohibiting him/her from responding. In the case of a proxy, the relationship to the sample adult is obtained. Of the 26,742 adults included in the NHIS in 2017, only in 423 cases or 1.58% cases, a knowledgeable proxy answered for the sample adult. Of these 423 cases, in 367 (87%) cases the proxy was a relative who lived in the same household.

Source: National Center for Health Statistics. Survey Description, National Health Interview Survey, 2017. Hyattsville, Maryland. 2018.

eFigure 1. Selection of Study Participants

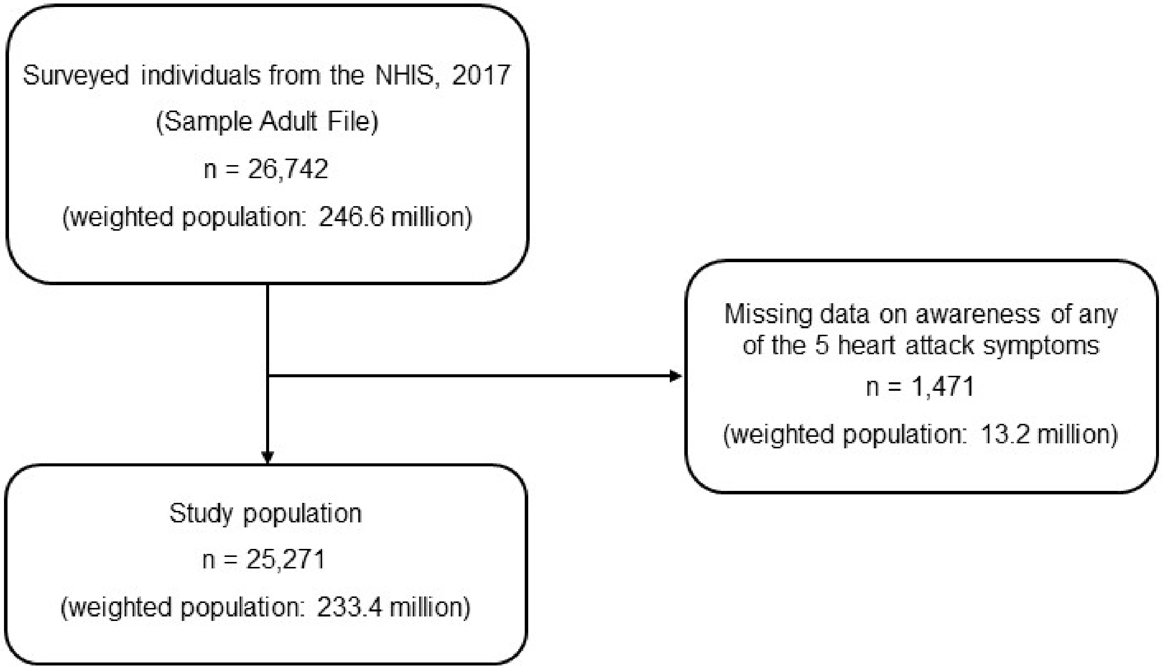

© 2019 Mahajan S et al. *JAMA Network Open.*

eFigure 2. Distribution of Awareness of Myocardial Infarction Symptoms by Sociodemographic Characteristics

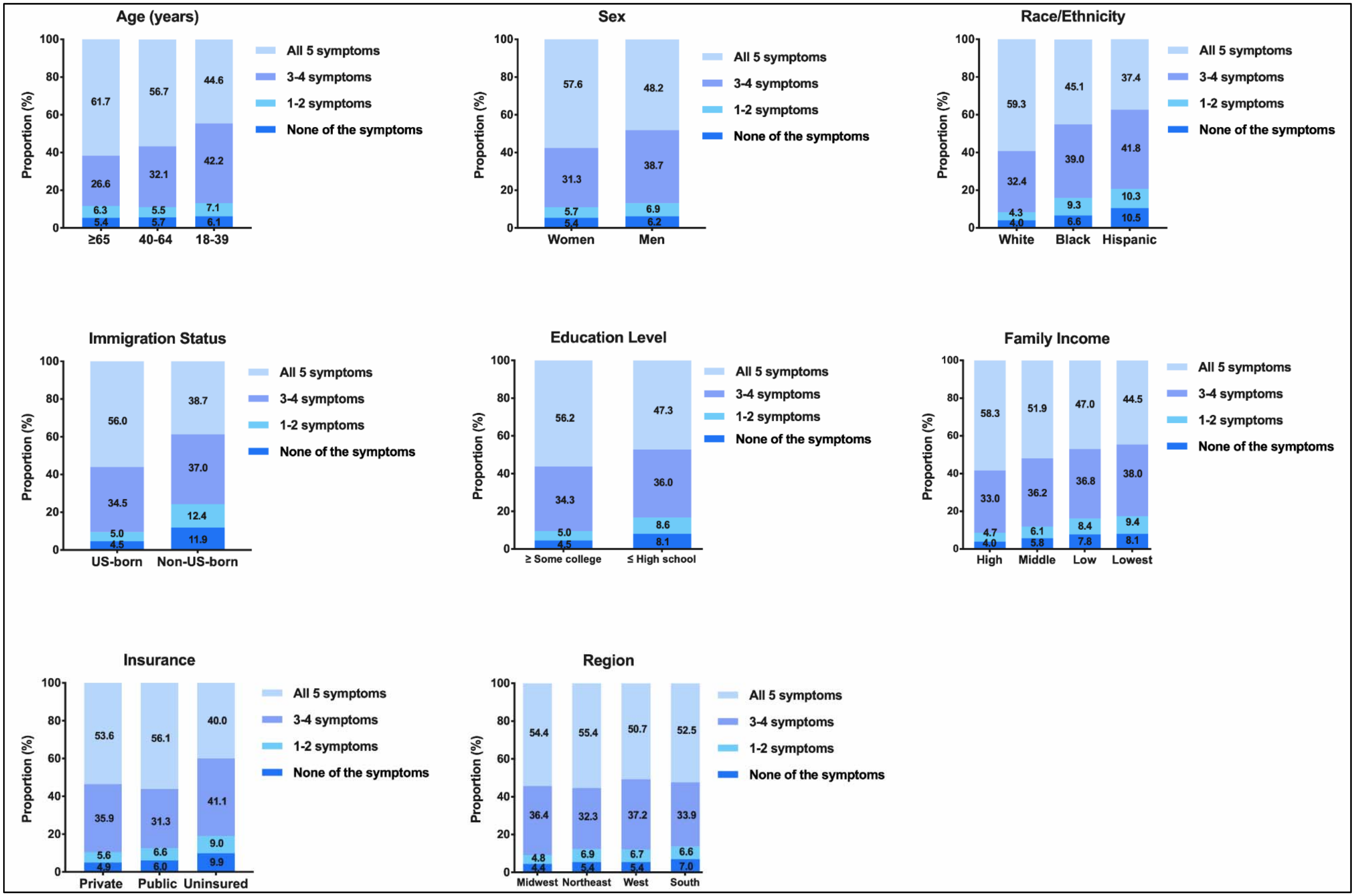

Abbreviations: US, United States

© 2019 Mahajan S et al. *JAMA Network Open.*

eFigure 3. Weighted Proportion of Individuals Who Were Not Aware of Any Myocardial Infarction Symptoms by Sociodemographic Characteristics

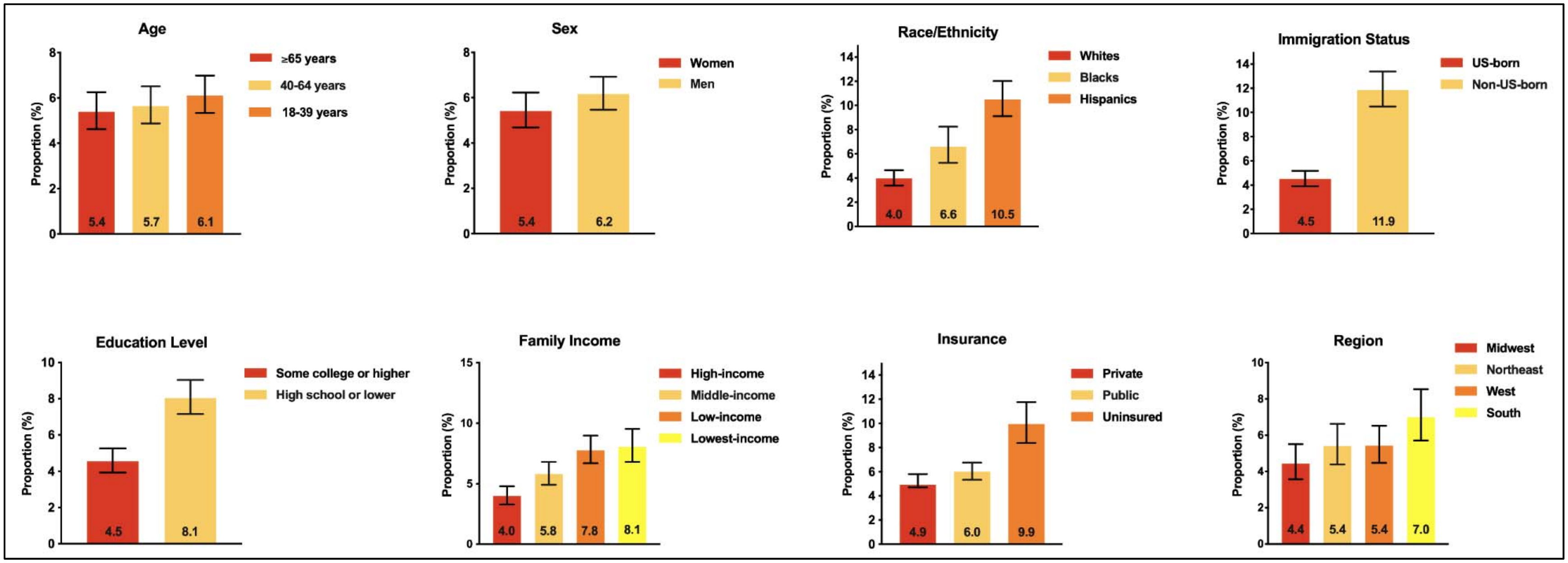

Abbreviations: US, United States

© 2019 Mahajan S et al. *JAMA Network Open.*

eFigure 4. Distribution of Awareness of Myocardial Infarction Symptoms and the Weighted Proportion of Non-US-Born Individuals Who Were Not Aware of Any Myocardial Infarction Symptoms, by English Proficiency and Years in the United States

A. Distribution of awareness of myocardial infarction symptoms among non-US-born individuals.

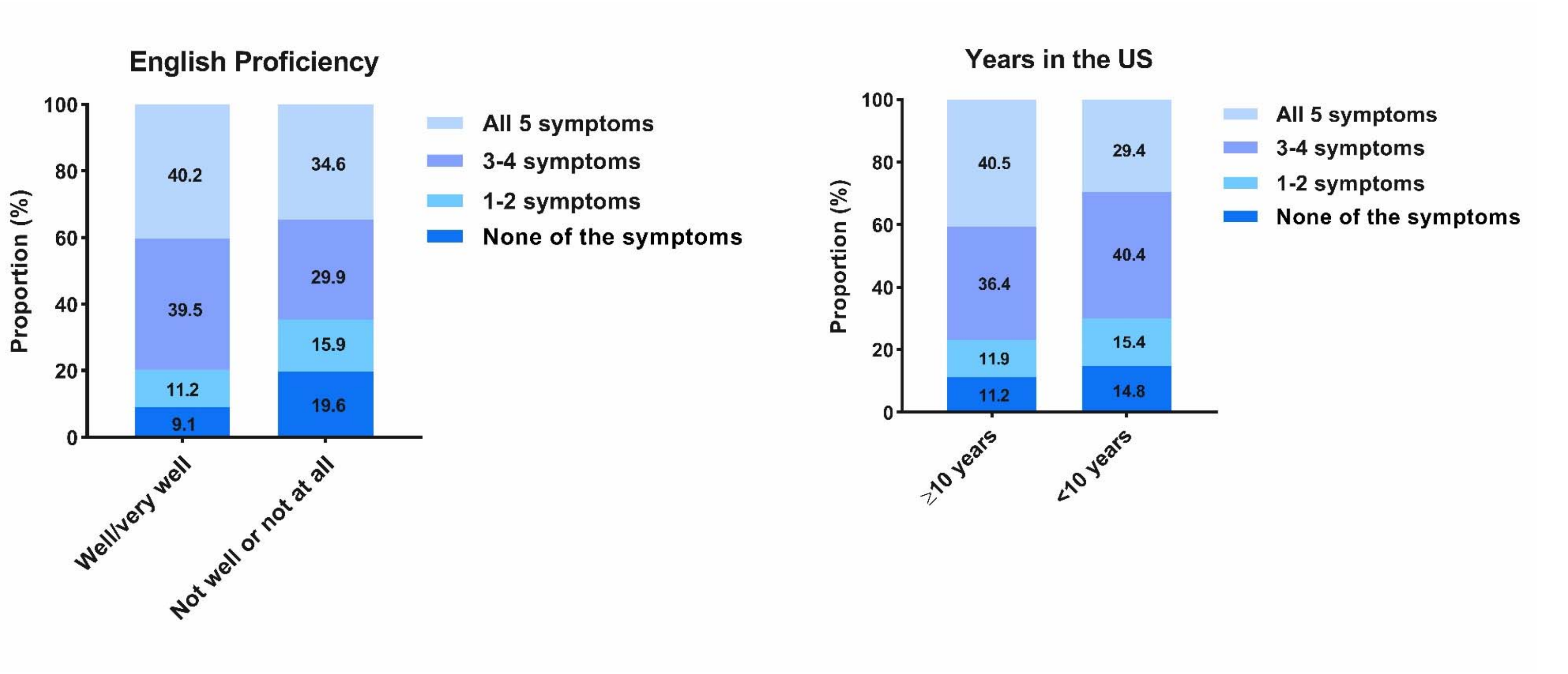

© 2019 Mahajan S et al. *JAMA Network Open.*

B. Weighted proportion of non-US-born individuals who were not aware of any myocardial infarction symptoms.

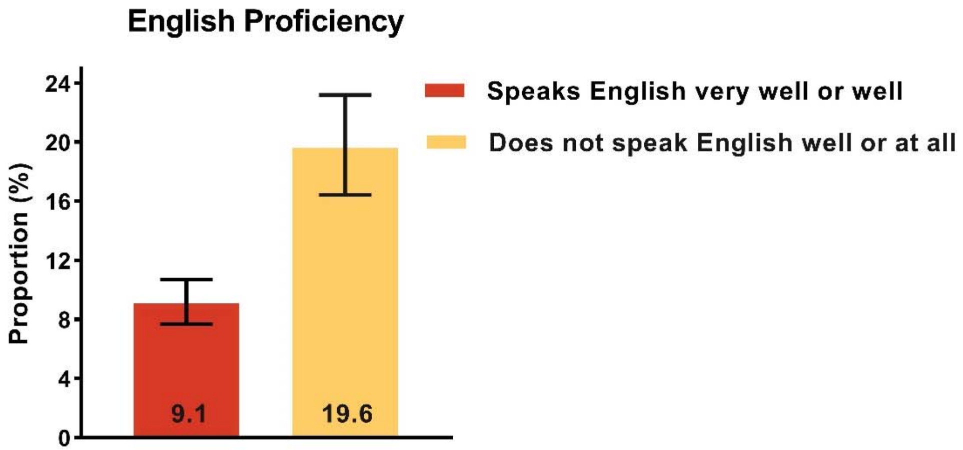

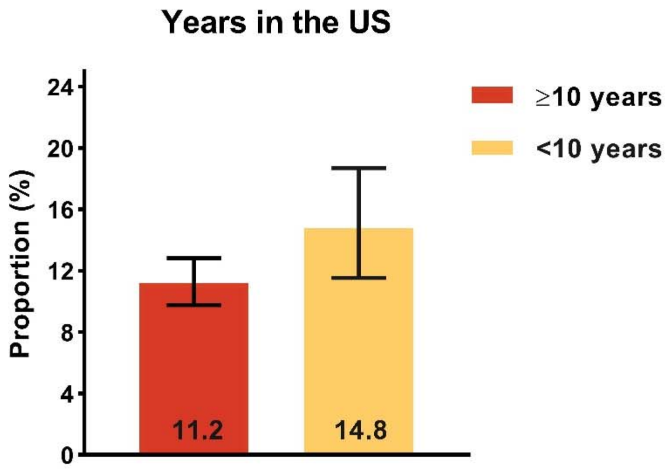

© 2019 Mahajan S et al. *JAMA Network Open.*

eTable 1. Awareness of Individual Symptoms of a Myocardial Infarction, by Sociodemographic Characteristics

Characteristics	Chest pain or discomfort		Shortness of breath		Pain or discomfort in arm		Feeling weak, lightheaded, or faint		Jaw, neck, or back pain	
	No. aware	% Aware (95% CI)	No. aware	% Aware (95% CI)	No. aware	% Aware (95% CI)	No. aware	% Aware (95% CI)	No. aware	% Aware (95% CI)
**Overall**	23,383	91.8 (91.0-92.6)	22,158	87.0 (86.1-87.8)	22,064	85.7 (84.8-86.5)	19,760	77.0 (76.1-77.9)	16,567	62.6 (61.6-63.7)
**Age, y**										
18-39	7,576	91.5 (90.4-92.5)	7,186	86.8 (85.7-87.9)	6,780	81.1 (79.8-82.4)	6,390	76.9 (75.5-78.2)	4,521	53.3 (51.8-54.9)
40-64	9,592	92.2 (91.3-93.1)	9,089	87.2 (86.1-88.1)	9,248	88.3 (87.3-89.3)	8,133	77.3 (76.1-78.5)	7,043	66.2 (64.9-67.5)
≥ 65	6,215	91.4 (90.3-92.5)	5,883	86.9 (85.7-88.0)	6,036	88.9 (87.8-90.0)	5,237	76.6 (75.1-78.0)	5,003	73.1 (71.7-74.6)
**Sex**										
Men	10,562	91.4 (90.5-92.3)	9,939	86.1 (85.0-87.0)	9,788	83.9 (82.7-85.0)	8,799	75.8 (74.7-76.9)	6,870	57.6 (56.3-58.9)
Women	12,821	92.1 (91.1-93.0)	12,219	87.9 (86.9-88.8)	12,276	87.4 (86.3-88.4)	10,961	78.1 (77.0-79.2)	9,697	67.3 (66.1-68.6)
**Race/Ethnicity**										
Non-Hispanic White	16,959	94.4 (93.5-95.1)	16,153	89.9 (89.0-90.7)	16,318	90.5 (89.7-91.3)	14,643	81.4 (80.5-82.3)	12,582	68.5 (67.4-69.5)
Non-Hispanic Black	2,525	90.5 (88.6-92.2)	2,364	85.1 (82.9-87.0)	2,267	79.1 (76.6-81.4)	1,995	72.0 (69.5-74.3)	1,591	55.7 (53.1-58.2)
Hispanic	2,529	84.8 (83.0-86.4)	2,356	79.5 (77.3-81.5)	2,331	76.9 (74.8-78.9)	2,000	66.3 (64.1-68.5)	1,498	46.8 (44.2-49.5)
**Immigration status**										
US-born	20,523	93.6 (92.8-94.4)	19,514	89.1 (88.2-89.9)	19,573	88.6 (87.8-89.4)	17,531	79.7 (78.8-80.6)	14,820	65.4 (64.3-66.4)
Non-US-born	2,843	83.1 (81.4-84.6)	2,629	77.2 (75.1-79.1)	2,474	71.6 (69.4-73.7)	2,217	64.0 (61.7-66.2)	1,739	49.6 (47.1-52.2)
**Education**										
Some college or higher	15,566	93.7 (92.9-94.4)	14,823	89.0 (88.1-89.8)	14,774	88.4 (87.5-89.2)	13,427	80.3 (79.3-81.3)	11,269	65.4 (64.2-66.6)
High school or lower	7,755	88.5 (87.2-89.6)	7,278	83.3 (82.1-84.5)	7,233	80.8 (79.3-82.1)	6,285	71.2 (69.8-72.5)	5,257	57.8 (56.3-59.2)

© 2019 Mahajan S et al. *JAMA Network Open.*

eTable 1. Continued.

**Family income**										
High-income	9,124	94.4 (93.4-95.2)	8,728	90.0 (88.9-90.9)	8,726	89.7 (88.7-90.6)	7,905	81.1 (79.8-82.3)	6,694	67.1 (65.6-68.5)
Middle-income	6,264	91.7 (90.6-92.7)	5,914	86.4 (85.1-87.7)	5,952	86.0 (84.6-87.3)	5,297	76.9 (75.4-78.3)	4,481	62.1 (60.4-63.7)
Low-income	3,782	88.8 (87.4-90.1)	3,552	83.6 (82.0-85.0)	3,526	81.0 (79.3-82.6)	3,139	72.9 (71.2-74.6)	2,569	57.2 (55.2-59.2)
Lowest-income	2,875	87.7 (85.7-89.5)	2,697	83.1 (81.2-84.8)	2,590	77.8 (75.2-80.2)	2,326	70.6 (68.3-72.8)	1,853	55.3 (52.7-57.8)
**Insurance**										
Private insurance	12,002	93.2 (92.2-94.1)	11,409	88.3 (87.3-89.3)	11,282	87.1 (86.1-88.1)	10,254	79.0 (77.8-80.1)	8,266	62.5 (61.2-63.8)
Public Insurance	9,424	91.0 (90.0-91.9)	8,916	86.4 (85.4-87.4)	9,034	86.6 (85.6-87.6)	7,928	76.0 (74.8-77.2)	7,128	66.6 (65.4-67.8)
Uninsured	1,884	86.4 (84.3-88.2)	1,766	81.4 (79.1-83.6)	1,687	74.4 (71.6-77.1)	1,520	69.2 (66.6-71.6)	1,136	50.1 (47.3-52.9)
**Region**										
Northeast	3,817	92.4 (91.0-93.6)	3,594	86.9 (85.2-88.5)	3,589	86.0 (84.1-87.7)	3,197	77.8 (75.6-79.8)	2,735	64.4 (62.1-66.6)
Midwest	5,687	93.9 (92.7-94.9)	5,415	89.1 (87.6-90.4)	5,391	88.3 (86.9-89.6)	4,843	79.2 (77.4-81.0)	4,045	64.8 (63.0-66.5)
South	8,520	90.4 (88.6-91.9)	8,070	85.5 (83.7-87.1)	8,054	84.2 (82.2-86.0)	7,147	75.5 (73.9-77.0)	6,048	61.9 (60.0-63.8)
West	5,359	91.6 (89.8-93.0)	5,079	87.4 (86.0-88.7)	5,030	85.3 (83.7-86.8)	4,573	76.6 (74.6-78.5)	3,739	60.3 (58.1-62.5)

Note: % aware represents the weighted proportion of adults who were aware of that symptom.
Abbreviations: CI, Confidence Interval; US, United States

© 2019 Mahajan S et al. *JAMA Network Open.*

eTable 2. Awareness of Symptoms of a Myocardial Infarction (0 to 5), by Sociodemographic Characteristics

Characteristics	None of the 5		1 of the 5		2 of the 5		3 of the 5		4 of the 5		All 5	
	n	Weighted %	n	Weighted %	n	Weighted %	n	Weighted %	n	Weighted %	n	Weighted %
**Overall**	1295	5.77%	483	1.97%	990	4.32%	2618	11.32%	5810	23.61%	14,075	53.01%
**Age (years)**												
18-39	454	6.11%	150	1.76%	382	5.37%	1094	14.40%	2333	27.82%	3785	44.54%
40-64	499	5.64%	181	2.00%	342	3.52%	958	9.91%	2254	22.22%	6070	56.71%
≥65	342	5.39%	152	2.29%	266	4.00%	566	8.29%	1223	18.32%	4220	61.72%
**Sex**												
Men	624	6.16%	223	1.97%	544	4.98%	1354	12.88%	2945	25.84%	5761	48.17%
Women	671	5.41%	260	1.96%	446	3.71%	1264	9.85%	2865	21.51%	8314	57.57%
**Race/Ethnicity**												
Non-Hispanic White	653	3.97%	224	1.30%	515	3.01%	1549	8.84%	4091	23.60%	10,878	59.27%
Non-Hispanic Black	164	6.60%	77	2.41%	193	6.89%	395	15.37%	671	23.62%	1282	45.11%
Hispanic	331	10.47%	126	3.73%	180	6.63%	450	16.82%	737	24.97%	1186	37.39%
**Immigration status**												
US-born	877	4.50%	324	1.40%	735	3.60%	2091	10.26%	5101	24.22%	12,698	56.01%
Non-US-born	418	11.87%	159	4.67%	254	7.77%	523	16.31%	704	20.66%	1370	38.73%
**Education**												
Some college or higher	667	4.55%	215	1.36%	539	3.60%	1529	10.05%	3856	24.22%	9711	56.23%
High school or lower	623	8.04%	263	2.97%	447	5.66%	1078	13.48%	1943	22.53%	4329	47.31%
**Family income**												
High-income	339	3.98%	126	1.48%	273	3.21%	818	9.43%	2189	23.57%	5859	58.34%
Middle-income	334	5.79%	96	1.75%	252	4.40%	695	11.55%	1577	24.65%	3783	51.86%
Low-income	285	7.76%	117	2.64%	219	5.81%	495	12.92%	982	23.85%	2120	47.02%
Lowest-income	222	8.06%	116	3.56%	183	5.81%	456	15.52%	764	22.53%	1487	44.52%
**Insurance**												
Private insurance	547	4.93%	173	1.63%	446	3.99%	1290	10.83%	3167	25.08%	7122	53.54%
Public Insurance	534	6.00%	223	1.99%	436	4.62%	992	10.23%	2086	21.06%	6003	56.10%
Uninsured	211	9.94%	84	3.79%	103	5.19%	316	16.94%	540	24.13%	919	40.00%

© 2019 Mahajan S et al. *JAMA Network Open.*

eTable 2. Continued.

Characteristics	None of the 5		1 of the 5		2 of the 5		3 of the 5		4 of the 5		All 5	
	n	Weighted %	n	Weighted %	n	Weighted %	n	Weighted %	n	Weighted %	n	Weighted %
**Region**												
Northeast	192	5.40%	95	2.38%	175	4.55%	408	10.12%	902	22.16%	2331	55.40%
Midwest	245	4.44%	83	1.44%	189	3.34%	582	10.35%	1511	26.06%	3426	54.37%
South	588	6.99%	204	2.11%	394	4.51%	1001	11.67%	2051	22.24%	5128	52.48%
West	270	5.42%	101	1.92%	232	4.77%	627	12.61%	1346	24.59%	3190	50.70%

Abbreviations: US, United States.

© 2019 Mahajan S et al. *JAMA Network Open.*

eTable 3. Proportion of Individuals Who Were not Aware of the 3 Most Common Symptoms of a Myocardial Infarction, by Sociodemographic Characteristics

3 most common symptoms include: chest pain/discomfort; pain/discomfort in arms/shoulders; and shortness of breath

Characteristics	n	Weighted % (95% CI)	US population (n)
**Overall**	4698	20.33 (19.38 - 21.32)	47,454,926
**Age (years)**			
18-39	1859	24.70 (23.31 - 26.13)	22,168,989
40-64	1650	17.58 (16.50 - 18.72)	17,196,499
≥65	1189	17.65 (16.38 - 18.99)	8,089,438
**Sex**			
Men	2396	22.57 (21.35 - 23.83)	25,511,966
Women	2302	18.23 (17.10 - 19.41)	21,942,960
**Race/Ethnicity**			
Non-Hispanic White	2541	14.76 (13.87 - 15.69)	22,401,380
Non-Hispanic Black	716	27.53 (25.04 - 30.18)	7,831,386
Hispanic	936	31.65 (29.36 - 34.02)	11,652,343
**Immigration status**			
US-born	3469	16.97 (16.05 - 17.92)	32,732,662
Non-US-born	1227	36.42 (34.23 - 38.67)	14,698,828
**Education**			
Some college or higher	2610	17.11 (16.15 - 18.12)	25,801,663
High school or lower	2067	26.13 (24.68 - 27.63)	21,365,049
**Family Income**			
High-income	1325	15.28 (14.18 - 16.45)	14,437,408
Middle-income	1209	20.82 (19.30 - 22.43)	12,985,918
Low-income	989	25.74 (24.01 - 27.56)	9,427,720
Lowest-income	879	29.43 (26.88 - 32.11)	7,510,476
**Insurance**			
Private insurance	2119	18.38 (17.21 - 19.61)	23,865,065
Public Insurance	1935	20.14 (19.07 - 21.26)	16,081,776
Uninsured	619	31.34 (28.70 - 34.11)	7,081,116
**Region**			
Northeast	764	20.02 (18.04 - 22.16)	8,579,151
Midwest	956	17.05 (15.45 - 18.79)	8,724,961
South	1880	21.80 (19.92 - 23.81)	18,533,609
West	1098	21.36 (19.72 - 23.09)	11,617,205
Abbreviations: CI, confidence intervals; US, United States.			

© 2019 Mahajan S et al. *JAMA Network Open.*

eTable 4. Association Between Population Characteristics and Awareness of Myocardial Infarction Symptoms Using Multinomial Regression Analysis

Characteristics	Aware of 1*	Aware of 2*	Aware of 3*	Aware of 4*	Aware of 5*
	RRR (95% CI)	RRR (95% CI)	RRR (95% CI)	RRR (95% CI)	RRR (95% CI)
**Sex**					
Men (Reference: Women)	0.90 (0.67 - 1.21)	1.08 (0.85 - 1.36)	1.08 (0.90 - 1.30)	0.95 (0.80 - 1.13)	0.67 (0.57 - 0.79) †
**Race/Ethnicity**					
Non-Hispanic Blacks (Reference: Non-Hispanic Whites)	1.06 (0.68 - 1.65)	1.52 (1.04 - 2.24) †	1.15 (0.79 - 1.68)	0.74 (0.52 - 1.05)	0.60 (0.43 - 0.84) †
Hispanics (Reference: Non-Hispanic Whites)	0.75 (0.47 - 1.18)	0.82 (0.54 - 1.24)	0.78 (0.58 - 1.04)	0.60 (0.46 - 0.79) †	0.42 (0.33 - 0.54) †
**Immigration status**					
Non-US-born (Reference: US-born)	1.29 (0.87 - 1.91)	0.91 (0.64 - 1.29)	0.70 (0.53 - 0.93) †	0.48 (0.37 - 0.62) †	0.46 (0.36 - 0.58) †
**Education**					
High school or lower (Reference: Some college or higher)	1.42 (1.05 - 1.93) †	1.08 (0.80 - 1.47)	0.94 (0.75 - 1.17)	0.73 (0.61 - 0.89) †	0.68 (0.56 - 0.82) †

*All compared to aware of none of the symptoms
† P-value <0.05
Abbreviations CI, Confidence Interval; RRR, Relative Risk Ratio; US, United States

© 2019 Mahajan S et al. *JAMA Network Open.*

eTable 5. Distribution of Different Responses to Assessment of Emergency Response to Suspicion of Myocardial Infarction

Responses	n	Weighted % (95% CI)
Call 911 or another emergency medical services number	24,088	95.54% (95.05 - 95.99)
Advise to drive to hospital	210	0.97% (0.76 - 1.23)
Advise to call physician	155	0.61% (0.47 - 0.79)
Call spouse or family member	99	0.39 (0.29 - 0.52)
Other	666	2.49 (2.21 - 2.80)
Abbreviations: CI, Confidence Interval.		

© 2019 Mahajan S et al. *JAMA Network Open.*

eTable 6. Proportion of Individuals Who Chose a Response Other Than Calling Emergency Medical Services in Response to Suspicion of a Myocardial Infarction, by Sociodemographic Characteristics

Other responses include - advise to call physician, drive to hospital, call spouse or family member, and other.

Characteristics	n	Weighted %	US population, n	p-value
**Overall**	1130	4.46	10,381,192	
**Age (years)**				0.001
18-39	285	3.95	3,542,792	
40-64	435	4.27	4,168,065	
≥65	410	5.84	2,670,335	
**Sex**				0.02
Women	590	4.09	4,909,591	
Men	540	4.85	5,471,601	
**Race/Ethnicity**				0.075
Non-Hispanic White	771	4.09	6,202,974	
Non-Hispanic Black	102	4.42	1,254,474	
Hispanic	162	5.50	2,014,323	
**Immigration status**				0.005
US-born	933	4.16	8,012,087	
Non-US-born	197	5.90	2,369,105	
**Education**				0.001
Some college or higher	671	3.91	5,888,368	
High school or lower	455	5.47	4,457,629	
**Family income**				<0.001
High-income	360	3.59	3,394,545	
Middle-income	293	4.36	2,711,562	
Low-income	221	5.42	1,980,933	
Lowest-income	165	5.87	1,490,372	
**Insurance**				<0.001
Private insurance	436	3.53	4,574,316	
Public Insurance	565	5.27	4,198,735	
Uninsured	125	6.82	1,536,767	
**Region**				0.452
Northeast	185	3.99	1,705,125	
Midwest	267	4.29	2,194,212	
South	424	4.89	4,149,487	
West	254	4.30	2,332,368	

Abbreviations: US, United States.

© 2019 Mahajan S et al. *JAMA Network Open.*

eTable 7. Association of Sociodemographic Characteristics With Choosing a Response Other Than Calling Emergency Medical Services on Suspicion of a Myocardial Infarction Using Logistic Regression

Other responses include - advise to call physician, drive to hospital, call spouse or family member, and other.

Characteristics	Unadjusted Model		Adjusted Model*	
	OR (95% CI)	*p-value*	*OR (95% CI)*	*p-value*
**Age (years)**				
18-39	Reference		Reference	
40-64	1.08 (0.88 - 1.34)	0.453	1.08 (0.85 - 1.38)	0.515
≥65	1.51 (1.19 - 1.91)	0.001	1.63 (1.22 - 2.19)	0.001
**Sex**				
Women	Reference		Reference	
Men	1.20 (1.03 - 1.40)	0.023	1.23 (1.04 - 1.46)	0.016
**Race/Ethnicity**				
Non-Hispanic White	Reference		Reference	
Non-Hispanic Black	1.08 (0.76 - 1.53)	0.650	0.99 (0.72 - 1.38)	0.968
Hispanic	1.36 (1.07 - 1.73)	0.010	1.15 (0.77 - 1.71)	0.493
**Immigration status**				
US born	Reference		Reference	
Non-US born	1.45 (1.12 - 1.88)	0.005	1.20 (0.81 - 1.78)	0.369
**Education**				
Some college or higher	Reference		Reference	
High school or lower	1.42 (1.19 - 1.70)	<0.001	1.22 (1.00 - 1.49)	0.046
**Family income**				
High-income	Reference		Reference	
Middle-income	1.22 (1.00 - 1.49)	0.046	1.05 (0.83 - 1.33)	0.049
Low-income	1.54 (1.21 - 1.95)	<0.001	1.16 (0.86 - 1.56)	0.339
Lowest-income	1.67 (1.22 - 2.29)	0.001	1.37 (1.00 - 1.88)	0.679
**Insurance**				
Private insurance	Reference		Reference	
Public Insurance	1.52 (1.28 - 1.81)	<0.001	1.11 (0.86 - 1.44)	0.434
Uninsured	2.00 (1.49 - 2.69)	<0.001	1.59 (1.19 - 2.12)	0.001
**Region**				
Midwest	Reference		Reference	
Northeast	0.93 (0.68 - 1.25)	0.615	1.07 (0.77 - 1.48)	0.691
South	1.15 (0.85 - 1.54)	0.365	1.06 (0.79 - 1.42)	0.702
West	1.00 (0.77 - 1.30)	0.992	0.99 (0.74 - 1.34)	0.954

*Model adjusted for all population characteristics
Abbreviations: OR, odds ratios; CI, confidence intervals; US, United States.

© 2019 Mahajan S et al. *JAMA Network Open.*

eTable 8. Proportion of Individuals Who Chose a Response Other Than Calling Emergency Medical Services in Response to Suspicion of a Myocardial Infarction, by Sociodemographic Characteristics and Awareness of Myocardial Infarction Symptoms

Characteristics	Aware of none of the symptoms		Aware of all 5 symptoms	
	n	Weighted % (95% CI)	n	Weighted % (95% CI)
**Overall**	115	9.79 (7.69 - 12.40)	538	3.44 (3.07 - 3.85)
**Age (years)**				
18-39	43	11.15 (7.84 - 15.62)	89	2.30 (1.77 - 2.99)
40-64	34	7.86 (5.19 - 11.75)	218	3.35 (2.85 - 3.93)
≥65	38	11.03 (7.42 - 16.07)	231	5.24 (4.51 - 6.07)
**Sex**				
Women	60	9.60 (6.20 - 14.56)	307	3.23 (2.80 - 3.72)
Men	55	9.98 (7.46 - 13.23)	231	3.71 (3.16 - 4.36)
**Race/Ethnicity**				
Non-Hispanic White	43	7.47 (4.69 - 11.70)	414	3.43 (3.03 - 3.88)
Non-Hispanic Black	15	13.71 (7.50 - 23.74)	42	2.69 (1.84 - 3.93)
Hispanic	40	10.42 (7.32 - 14.63)	43	3.83 (2.61 - 5.61)
**Immigration status**				
US-born	66	8.94 (6.46 - 12.24)	490	3.48 (3.09 - 3.93)
Non-US-born	49	11.39 (8.45 - 15.17)	48	3.16 (2.21 - 4.51)
**Education**				
Some college or higher	45	7.64 (5.45 - 10.61)	360	3.34 (2.94 - 3.80)
High school or lower	69	11.97 (8.94 - 15.84)	176	3.66 (3.01 - 4.44)
**Family income***				
High/middle-income	51	7.63 (5.27 - 10.93)	343	3.18 (2.77 - 3.65)
Low/lowest-income	55	12.50 (9.19 - 16.79)	155	4.19 (3.44 - 5.11)
**Insurance**				
Private insurance	50	8.21 (5.03 - 13.11)	311	2.48 (2.09 - 2.93)
Public Insurance	39	10.43 (7.50 - 14.33)	192	4.93 (4.28 - 5.69)
Uninsured	26	13.14 (8.44 - 19.89)	35	3.63 (2.34 - 5.59)
**Region**				
Northeast	12	5.93 (2.70 - 12.51)	102	3.60 (2.73 - 4.73)
Midwest	16	8.92 (4.93 - 15.62)	125	3.25 (2.61 - 4.04)
South	61	11.07 (7.69 - 15.70)	180	3.27 (2.72 - 3.92)
West	26	10.94 (7.19 - 16.29)	131	3.79 (2.93 - 4.88)

Abbreviations: CI, confidence interval; US, United States
*We combined high-income and middle-income groups into high/middle-income, and low-income and lowest-income groups into low/lowest-income due to small sample sizes.

© 2019 Mahajan S et al. *JAMA Network Open.*
